# Inhibited Endogenous H_2_S Generation and Excessive Autophagy in Hippocampus Contribute to Sleep Deprivation-Induced Cognitive Impairment

**DOI:** 10.3389/fpsyg.2019.00053

**Published:** 2019-01-24

**Authors:** San-Qiao Yang, Li Jiang, Fang Lan, Hai-jun Wei, Ming Xie, Wei Zou, Ping Zhang, Chun-Yan Wang, Yu-Rong Xie, Xiao-Qing Tang

**Affiliations:** ^1^Institute of Neuroscience, Hengyang Medical College, University of South China, Hengyang, China; ^2^Department of Neurology, Affiliated Nanhua Hospital, University of South China, Hengyang, China; ^3^Department of Neurology, First Affiliated Hospital of University of South China, Hengyang, China; ^4^College of Chemistry and Chemical Engineering, University of South China, Hengyang, China

**Keywords:** autophagy, cognitive impairment, hydrogen sulfide, sleep deprivation, hippocampus

## Abstract

**Background and Aim:** Sleep deprivation (SD) causes deficit of cognition, but the mechanisms remain to be fully established. Hydrogen sulfide (H_2_S) plays an important role in the formation of cognition, while excessive and prolonged autophagy in hippocampus triggers cognitive disorder. In this work, we proposed that disturbances in hippocampal endogenous H_2_S generation and autophagy might be involved in SD-induced cognitive impairment.

**Methods:** After treatment of adult male wistar rats with 72-h SD, the Y-maze test, object location test (OLT), novel object recognition test (NORT) and the Morris water maze (MWM) test were performed to determine the cognitive function. The autophagosome formation was observed with electron microscope. Generation of endogenous H_2_S in the hippocampus of rats was detected using unisense H_2_S microsensor method. The expressions of cystathionine-β-synthase (CBS), 3-mercaptopyruvate sulfurtransferase (3-MST), beclin-1, light chain LC3 II/LC3 I, and p62 in the hippocampus were assessed by western blotting.

**Results:** The Y-maze, OLT, NORT, and MWM test demonstrated that SD-exposed rats exhibited cognitive dysfunction. SD triggered the elevation of hippocampal autophagy as evidenced by enhancement of autophagosome, up-regulations of beclin-1 and LC3 II/LC3 I, and down-regulation of p62. Meanwhile, the generation of endogenous H_2_S and the expressions of CBS and 3-MST (H_2_S producing enzyme) in the hippocampus of SD-treated rats were reduced.

**Conclusion:** These results suggested that inhibition of endogenous H_2_S generation and excessiveness of autophagy in hippocampus are involved in SD-induced cognitive impairment.

## Introduction

Sleep plays a key role in human life and work, but sleep deprivation (SD), namely irregular and inadequate sleep, shows a rising trend in today’s society (Feng et al., 2016; Honn et al., 2018). Therefore, the association between SD and cognitive impairment has been paid close attention (Zhang and Liu, 2008; Jin et al., 2017; Li S. et al., 2017). It is well known that SD affects human health and work efficiency (Honn et al., 2018) and that SD is a common state leading to a global cognitive decline for individuals (Jackson et al., 2013). Meanwhile, extensive studies confirmed that rats deprived of sleep is embodied in various morphological and neurobiological changes in the brain and a decline of cognitive behavior (Zhang L. et al., 2013; Zhao et al., 2014; Hajali et al., 2015; Kreutzmann et al., 2015). Recent studies showed that the oxidative stress and the disorder of neurotransmitters play important roles in the cognitive impairment induced by SD (Lu et al., 2018; Nabaee et al., 2018; Siddique et al., 2018). In addition, increasing evidence showed that SD impacts the epigenome that plays an important role in regulating learning and memory (Duan et al., 2016; Gaine et al., 2018). Although the research of cognitive impairment induced by SD has been reported, further exploring the mechanism underlying SD-induced cognitive impairment is necessary for a greater understanding of the pathophysiology about SD and its effects on cognition.

Hydrogen sulfide (H_2_S) is a colorless gas with the smell of rotten eggs and was considered toxic in the past (Reiffenstein, 1992). In recent years, it has been confirmed that H_2_S is an important neuroprotective and neuromodulatory agent (Bae et al., 2013; Zhang and Bian, 2014; Jiang et al., 2016). Endogenous H_2_S is largely synthesized in mammalian tissues by cystathionine-β-synthase (CBS) in the brain and 3-mercaptopyruvate sulfurtransferase (3-MST) in the mitochondria (Kimura, 2011). It has been demonstrated that H_2_S promotes the formation of long-term potentiation (LTP) (Chen et al., 2017), improves synaptic plasticity remodeling (Li Y. L. et al., 2017), and regulates learning and memory (Whiteman et al., 2011; Li M. et al., 2017; Tang et al., 2018; Zhan et al., 2018). Furthermore, our previous work has confirmed that inhibition of H_2_S synthesis contributes to formaldehyde- and homocysteine-induced defects in learning and memory of rats (Tang X. Q. et al., 2013; Li M.H. et al., 2014). Therefore, we investigated whether the inhibition of hippocampal endogenous H_2_S generation is responsible for SD-impaired learning and memory.

Autophagy is a catabolic process that digests the useless cytosolic components through invagination of its membrane in order to maintain cell homeostasis and integrity (Barthet and Ryan, 2018; Sharma et al., 2018). Studies demonstrated that the level of autophagy is regulated by a variety of factors, such as oxidative stress, energy balance or aging (Zhang et al., 2016; Loos et al., 2017). However, the change of autophagy level under conditions of SD remains unknown. Accumulating evidence suggests that the disorder of hippocampal autophagy plays crucial role in the formation of cognitive dysfunction in neurological diseases (Ghavami et al., 2014), including Parkinson’s disease (PD) (Zhang S. et al., 2013) and Alzheimer’s disease (AD) (Zheng et al., 2006; Sharma et al., 2018). Therefore, we speculated that excessive hippocampal autophagy is injurious to the cognitive function of SD-exposed rats.

The present work was to clarify the relationship between hippocampal endogenous H_2_S generation as well as autophagy and SD-induced cognitive impairment. We demonstrated that exposure of SD impaired the function of cognition, suppressed the generation of hippocampal H_2_S, and stimulated the excessiveness of hippocampal autophagy. These results suggest that inhibited endogenous H_2_S generation and excessive autophagy in hippocampus play important role in SD-induced cognitive impairment.

## Materials and Methods

### Reagents

Specific monoclonal anti-LC3, anti-Beclin1, and anti-p62 antibodies were obtained from Cell Signaling Technology, Inc. (Danvers, MA, United States). Specific monoclonal anti-CBS and anti-3-MST were purchased from Santa Cruz Biotechnology, Inc. (Santa Cruz, CA, United States).

### Animals and Experiment Schedule

Adult male Wistar rats (weighing 250–280 g) were purchased from the SJA Lab Animal Center of Changsha (Changsha, China), were housed individually in a constant temperature (25 ± 2°C) and humidity controlled room, the rats were illuminated with artificial light for 12-h light/12-h dark cycle and access to food and water *ad libitum*. Experimental protocol of the study was approved by the Animal Use and Protection Committee of the University of South China. Rats were used according to “3Rs” principles (Replacement, Reduction and Refinement) in all experimental procedures. All efforts were made to minimize animal suffering.

All rats were habituated to the experimenter and laboratory for 1 week pre-handled prior to testing. SD group rats were exposed to the modified multiple platform method (MMPM) for 72 h. The Y-maze was performed one day after SD. The NORT was performed 2 days after Y-maze test. The OLT was performed 2 days after NORT. The MWM test was performed 2 days after OLT. One day after behavioral testing, the hippocampal tissues were collected for detecting the generation of H_2_S and the level of autophagy.

### Induction of Sleep Deprivation

The method of SD was adopted from the modified multiple platform method. Animals were exposed to the modified multiple platform method (MMPM) for 72 h. In brief, rats were placed on platforms (19 platforms; 6.5 cm in diameter, 15 cm apart edge-to-edge) surrounded by water (24 ± 1°C) which were located 1 cm below the water surface in a water tank (170 cm × 70 cm × 50 cm) where water and food were accessible. The method was used to disturb the total sleep (especially REM sleep). During REM sleep, muscle atonia caused animals to fall into or touch the water and waken. Immediately after SD, animals were submitted to behavioral tasks.

### Behavioral Testing

#### Y-Maze

The Y-maze apparatus is composed of 3 arms with identical dimensions (120°; 55 cm long × 16 cm wide × 20 cm high),was placed inside a room with dim illumination. The floor of the maze consists of sawdust to eliminate olfactory stimuli. Testing was always at the same time and performed in the same room to ensure environmental coincide. The rats prefer to explore a new arm of the maze rather than going back to a previous one. Briefly, each rat was inserted to the center of the Y-maze and allowed to explore freely the three arms during an 8-min session. A rat into an arm was considered valid if its body and tail completely entered the arm. The total number of arm entries and sequence were recorded with video and analyzed later on a computer. An alternation was identified as three consecutive entries in three different arms of the maze (i.e., 1, 2, 3, or 3, 2, 1, etc.). The alternation performance was calculated using the following equation: total alternation number/(total number of entries -2). At the same time, the total numbers of entries in the three arms were used to detecting the activity of rats.

#### Morris Water Maze

The Morris water maze system consists of water maze device, water maze image automatic collection and software analysis system. Pictures of mice swimming (analog signal) were collected via the camera was introduced to the computer for analog-to-digital conversion to get the relevant data through digital image analysis. This system is a classical program for evaluating the spatial learning and memory of rodents. Water maze device mainly consists of a circular pool containing water (diameter of 180 cm, high 60 cm) and a circular acrylic platform (12.5 cm diameter) placed 2 cm below the surface of the water during acquisition trials. The pool was divided into 4 quadrants, and platform was placed in the first quadrant (target quadrant). The video recorder was placed above the center of the pool, recording the experimental process and stored in a computer (Chengdu Technology and Market Corp, Chengdu, China). The rats were allowed four acquisition trials per day for 5 days conducted in a spaced fashion, and each rat was given a 120-s swim to find the platform. The swimming route and escape latencies to platform (s) were determined in each trial. Twenty-four hours after the last acquisition trial the rats were given a probe trail without the platform, and they were allowed a free 120-s swim to search for the pool. The start position for each animal was in contrast to the platform location and the platform quadrant was referred to as the target quadrant. The times of crossing former platform area and the proportionality of swimming time in target quadrant and Mid-ring were determined. In the visible-platform test, the platform was located 2 cm above the water surface. Swim speed was tested with a visible platform in the water maze to rule out the differences in performance could be owing to non-cognitive factors including stress (Lu et al., 2017) and depression (Yang et al., 2016).

#### Novel Object Recognition

The Novel object recognition test (NORT) surveys the exploration of familiar and novel objects, which is a part of recognition memory. The test consists of three stages: adaptation, training and testing. During the adaptation period of 2 days, animals were habituated to the opaque empty square box (50 cm × 50 cm × 60 cm) for 5 min each day. In the training stage, the rat was placed into the testing box for 5 min to explore two different objects on opposite sides of the arena, the total approach time for exploring each object were recorded by an experienced researcher blind to treatments. The following acts were considered as the exploration of the object: touching to the object with the head of animal, sniffing to the object and keeping the distance from nose of rat to the objects less than 2 cm. The apparatus include testing box and the objects were cleaned with 70% alcohol at the end of each experiment for every rat. During testing sessions, one familiar object and one novel object of similar size were placed into the same places as in the training phase, and the animals were permitted to explore for 5 min. Calculation of the object recognition memory by measuring the interest in the novel object in testing phase which is called recognition index (RI) was expressed as the time exploring on the novel objects divided by the total time spent in exploring both objects.

#### Object Location

The Object location test (OLT) following with the same rules on NORT and consist of three identical parts (adaptation, training, and testing). The difference between OLT and NORT is that the exploration time in measuring two same objects but one of them was placed in a new position, also the recognition index (RI) is similar to NORT was calculated as the time exploring on the objects placed in novel location divided by the total time spent in exploring both objects. It evaluates especially spatial memory and discrimination.

### Transmission Electron Microscopy (TEM)

Transmission electron microscopy was used to assess the ultrastructural change of hippocampus sections. The volume of hippocampus fragments for electron microscopy were obtained not more than 1 mm × 1 mm × 1 mm and then rapid fixed with 2.5% glutaraldehyde in 0.1 M PBS solution at 4°C for 2–4 h. Afterward, they were washed three times for 15 min each with PBS and then fixed with 1% osmic acid for 2 h. After washed with PBS, tissues were dehydrated in a graded ethanol series, embedded in Epo × 812 overnight. Ultra-thin sections were cut at 60–80 nm thickness and double colored with uranyl acetate and lead citrate, which were subsequently observed under a 1230 type transmission electron microscope (Electron Co., Tokyo) and photographed.

### Western Blotting Analysis

The hippocampal tissues were homogenized in extraction buffer (50 mM Tris, pH 7.4, 1% Triton X-100, 150 mM NaCl, 1% sodium deoxycholate, 1 mM NaF, 0.1% SDS, 2 mM Na3VO4, 1 mM PMSF) and then centrifuged at 12,000 rounds/min for 30 min at 4 °C. Protein concentrations were measured using a BCA Protein Assay Kit (Beyotime, Shanghai, China). Equivalent amounts of protein were run on SDS–PAGE (12% for LC3 and; 10% for CBS, 3-MST and Beclin1) and then transferred to a PVDF membrane. The membrane was blocked using TBS-T buffer (50 mM Tris–HCl, pH 7.5, 150 mM NaCl, 0.05% Tween-20) containing 5% non-fat milk at room temperature for 2 hr and then serially incubated with primary antibodies directed against CBS (1:2000), 3-MST (1:1000), LC3 (1:1000), Beclin1 (1:1000) and P62 (1:1000) overnight at 4°C. After washing with TBS-T three times for 10 min, respectively, the membrane was incubated with secondary antibody (1:5000) in blocking solution at room temperature for 2 h. The membrane was washed again for three times with TBS-T, the bound antibody was were visualized by autoradiographic films (Tanon-5600) and then tegrated optical density of the protein band from Western blot analyses was quantified using Image-J software. Each experiment was repeated at least three times.

### Assay of H_2_S Generation

To detect the generation of H_2_S in hippocampal tissue of rat, unisense H_2_S microsensor (a miniaturized amperometric sensor with guard electrode) (Model H_2_S-MRCh, Unisense, Aarhus, Denmark) coupled to a unisense picoampere amplifier was used. Hippocampus was homogenized in 50 mmol/L ice-cold potassium phosphate buffer (pH 6.8). After BCA quantitative analysis, the reaction mixture was added to the reaction bottle. The reaction mixture contained 100 mM potassium phosphate buffer (pH 7.4), L-cysteine (20 μl, 10 mM), pyridoxal 5′-phosphate (20 μl, 2 mM), 10% (w/v) tissue homogenate. Adding 1 mol/L NaOH to the central hole of the reaction bottle 0.5 mL, the reaction bottle is blown 20 s by N2 before sealing. Reaction was initiated by a thermostatic water bath for 90 min at 37°C. Then 50% (mass fraction) trichloroacetic acid was added into the reaction system. Finally, the reaction system was incubated at 37 C for 60 min to terminate the reaction. The concentration of H_2_S in the solution was determined by the sensitive sulfur electrode method in the central hole. The rate of H_2_S formation was calculated and expressed as nmol/(min × mg).

### Statistical Analysis

Data are expressed as the mean ± SEM. The significance of the difference between two groups was analyzed by the independent samples *t*-test with SPSS 20 software (SPSS, Chicago, IL, United States). Statistical significance was indicated at *p* < 0.05.

## Results

### SD Induces a Decrease in Alternation Performance in the Y-Maze Tested

**FIGURE 1:**
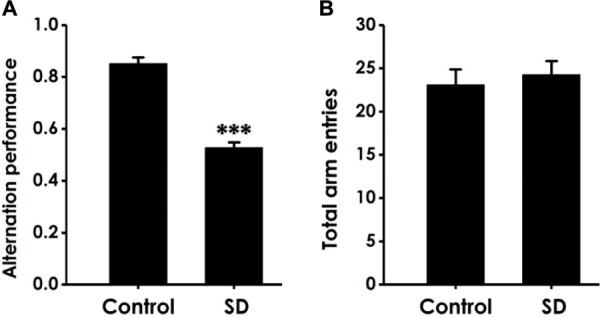
Effect of SD on the alternation performance of rats in Y male test. After exposure of SD for 72 h, rats were tested in the Y male test. The alternation performance **(A)** and the total arm entries **(B)** were recorded. The data are expressed as mean ± SEM (*n* = 9–11); ^∗∗∗^*P* < 0.001, vs. control group.

Y-maze test was subjected to detect whether the cognitive function of SD-exposed rats is impaired. As shown in Figure 1A, SD-exposed rats showed a significant decline in the alternation performance compared to control group. However, the total arm entries did not change between SD-rats and control group (Figure 1B). These data indicated that SD could impair learning and memory process of rats.

### SD Impairs the Cognitive Function of Rats in Novel Object Recognition (NOR) Test

**FIGURE 2:**
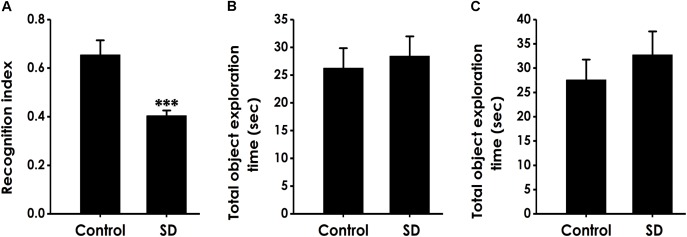
Effect of SD on the object recognition memory of rats. Rats were tested in the novel object recognition test. The recognition index in the test period **(A)** and the total object exploration time in the training period **(B)** or in the test period **(C)** were recorded. Values are the mean ± SEM (*n* = 9–11); ^∗∗∗^*P* < 0. 001, vs. control group.

Next, we used the novel object recognition test to examine the altheration of cognitive function in SD-exposed rats. As shown in Figure 2A, the recognition index in SD-exposed rats was markedly decreased compared to control in the test period. However, SD-exposed rats did not change the total object exploration time in the training period (Figure 2B) and the test period (Figure 2C). These data also indicated that SD impairs the cognitive function of rats.

### SD Causes Deficit in Location Memory in Object Location Test

**FIGURE 3:**
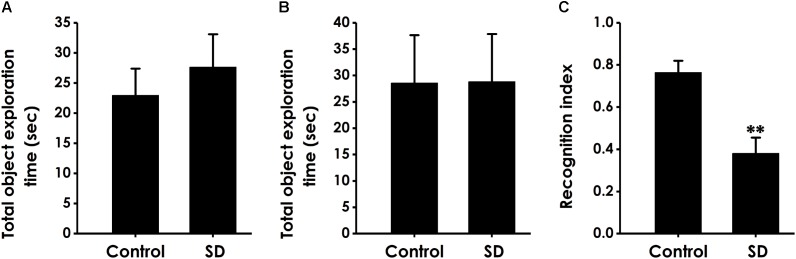
Effect of SD on the spatial recognition memory of rats. Rats were tested in the object location test. The total object exploration in the training period **(A)** and the total object exploration **(B)** as well as the recognition index **(C)** in the test period were recorded. Values are the mean ± SEM (*n* = 9–11); ^∗∗^*P* < 0.01, vs. control group.

We performed the object location test to extend our observation to a spatial form of cognition. SD did not affect the total object exploration time of rats in the training period (Figure 3A) and the test period (Figure 3B). However, compared to control groups, the recognition index in test period was significantly decreased in the rats treated with SD (Figure 3C), indicating that SD triggered deficit in object recognition memory.

### SD Impairs Learning and Memory in the Morris Water Maze Test

**FIGURE 4:**
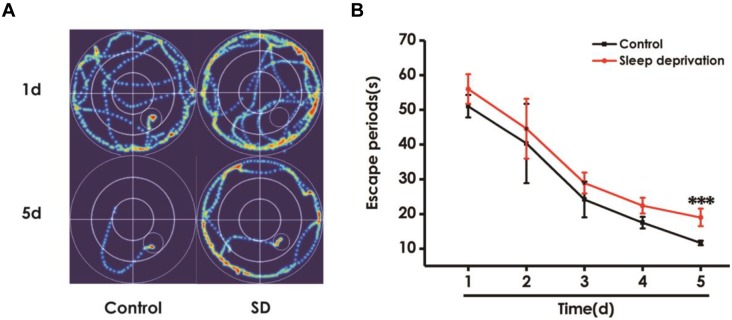
Effect of SD on the spatial learning in the acquisition phase of Morris water maze task. Rats were tested in the Morris water maze task. **(A)** The representative swimming route of rats in 1st day and 5th day in the acquisition phase. **(B)** The escape periods of rats in 1–5 days in the acquisition phase. Values are the mean ± SEM (*n* = 9–11), ^∗∗∗^*P* < 0.001, vs. control group.

To further explore the effect of SD on learning and memory in rats, we investigated the effects of SD on spatial learning and memory using the Morris water maze test. Figure 4A shows the representative swimming tracks of rats searching for the underwater platform on the first and fifth training days. On the first training day, there was no difference of the distance in searching for the hidden platform. On the fifth training day, SD-exposed rats exhibited a significant increase in the distance swam compared with the control group. Meanwhile, SD-treated rats exhibited significant higher escape latency on days 5 during training trials compared with control group rats (Figure 4B). These data further indicated that SD had an obvious negative effect on spatial learning of rats.

**FIGURE 5:**
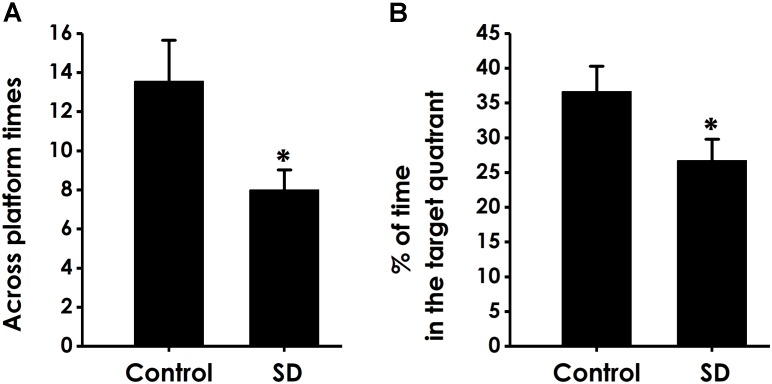
Effects of SD on the spatial memory in the probe phase of Morris water maze task. After finishing the acquisition phase, rats were submitted to the probe trial. The times of crossing platform **(A)** and the proportionality of swimming time in target quadrant **(B)** was analyzed. Values are the mean ± SEM (*n* = 9–11), ^∗^*P* < 0.05, vs. control group.

In the probe trial, the platform was removed and the rats were placed into the quadrant opposite to the target quadrant and allowed to swim freely for 120 s. Rats treated with SD exhibited significantly fewer number of crossing over the platform position (Figure 5A) and lower percentage of time in the target quadrant (Figure 5B) compared with control group, which indicated that SD impairs the spatial memory of rats.

### Ruling Out the Influences of the Changes in Motor Ability and Vision on Learning and Memory in Rats

**FIGURE 6:**
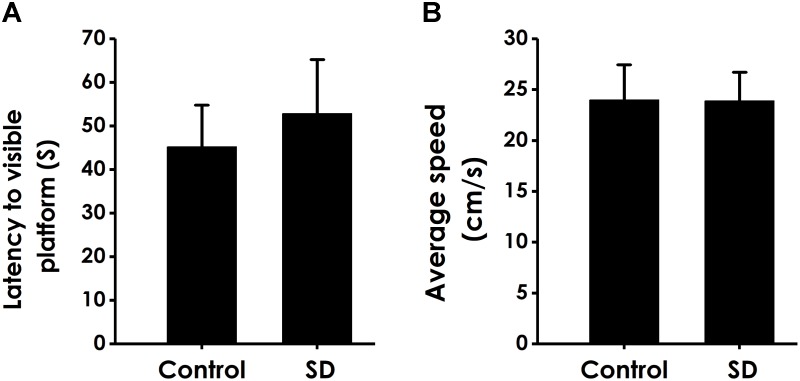
Effects of SD on the vision and motion of rats. After finishing the probe test, rats were submitted to the visible platform test. The latency to reach the platform **(A)** and the average swimming speed **(B)** were recorded. Values are the mean ± SEM (*n* = 9–11).

In order to exclude possible changes in visual acuity and motor ability, we tested the escape latency and the average swimming speed of rats in the visual platform test after we completed the probe test. There was no significant difference in the escape latency (Figure 6A) and the average swimming speed (Figure 6B) among all rats, which ruled out the possible that the alters in vision and motion contribute to the changes of all parameters in MWM experiment.

### SD Causes Excessive Autophagy in Hippocampus of Rats

**FIGURE 7:**
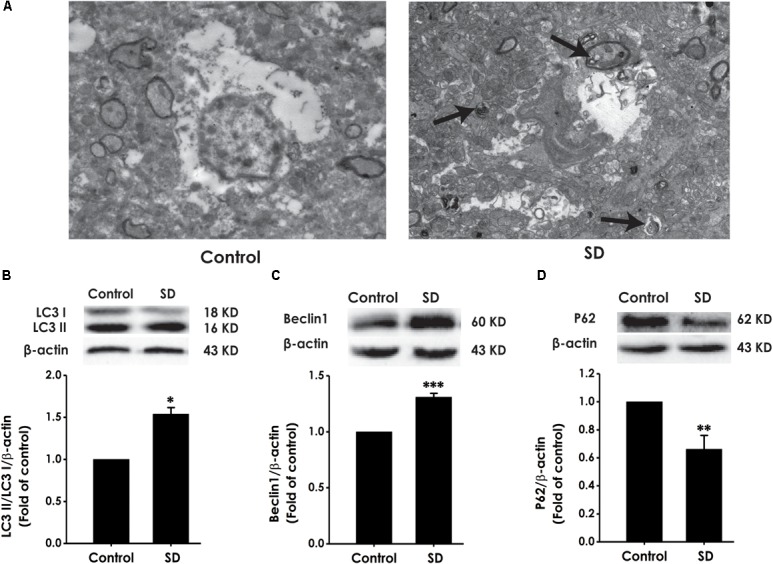
Effects of SD on the autophagy in the hippocampus of rats. After 72 h exposure to SD, the rat’s hippocampus was separated. Autophagic vacuoles **(A)** was observed under transmission electron microscope. Arrows indicate autolysosome-like vesicles in the cytoplasm. LC3-II/LC3-I **(B)**, Beclin-1 **(C)**, and P62 **(D)** expressions in the hippocampus of rats were detected by Western blot using anti-LC3, -Beclin-1, and -P62 antibody, respectively. β-actin was used as loading control. Data are reported as the mean ± SEM (*n* = 3–5); ^∗^*P* < 0.05, ^∗∗^*P* < 0.0, ^∗∗∗^*P* < 0.001, vs. control group.

To explore whether excessive autophagy is involved in SD-induced impairment in cognition, the formation of autophagic vacuoles, as well as the expressions of LC3-II/LC3-I, Beclin-1 and P62 were investigated in the hippocampus of rat (Merenlender-Wagner et al., 2015). As shown in Figure 7A, SD-treated rats displayed increase in the formation of autophagic vacuoles. In addition, the ratio of LC3-II/LC3-I (Figure 7B) and the expression of Beclin-1 (Figure 7C) were significantly increased in the hippocampus of SD-exposed rats. While the expression of P62 was significantly down-regulated in the hippocampus of SD-treated rats (Figure 7D). These data indicated that SD exerts excressive autophagy in the hippocampus of rats.

### SD Reduces the Expressions of 3-MST, CBS and the Generation of H_2_S in the Hippocampus of Rats

**FIGURE 8:**
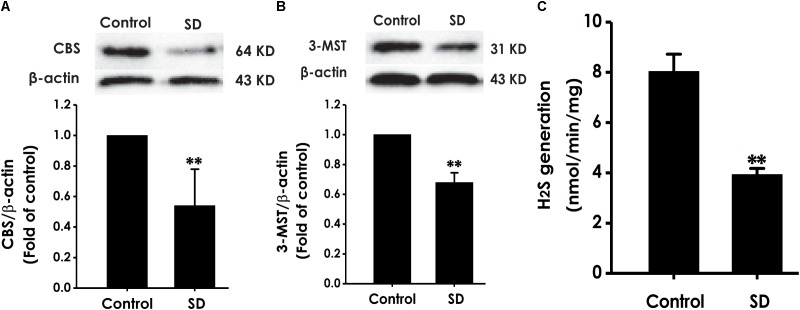
Effects of SD on the expressions of CBS and 3-MST and the generation of H_2_S in the hippocampus of rats. After 72 h SD treatment, the hippocampus of rats was homogenized. The expressions of CBS **(A)** and 3-MST **(B)** were measured by western blot analysis using anti-CBS and anti-3-MST antibody, respectively. β-actin was used as loading control. The generation of H_2_S **(C)** was analyzed by unisense H_2_S microsensor. Values are the mean ± SEM (*n* = 3–5); ^∗∗^*P* < 0.01 vs. control group.

To explore whether the inhibition of hippocampal H_2_S generation involves in SD-induced impairment in cognition, the expressions of CBS and 3-MST as well as the endogenous H_2_S generation in the hippocampus of rats were analyzed (Calvert et al., 2010). SD caused decrease in the expressions of CBS (Figure 8A) and 3-MST (Figure 8B) in the hippocampus of rats. Simultaneously, the endogenous H_2_S generation in the hippocampus of rats was significantly inhibited by treatment with SD for 72 h (Figure 8C). These data demonstrated that SD reduces the generation of endogenous H_2_S in the hippocampus of rats.

## Discussion

Sleep deprivation (SD) is considered as a common social phenomenon and is a frequent cause of cognitive impairment (Miller, 2015; Feng et al., 2016; Hao et al., 2018). It is well established that H_2_S regulates learning and memory (Nagpure and Bian, 2015; Zhuang et al., 2016; Tang et al., 2018) and that excessive autophagy in hippocampus causes cognitive impairment (Xu et al., 2016). Therefore, we explored the alterations in the hippocampal endogenous H_2_S generation and autophagy and the change of cognitive function in SD-exposed rats. Our present work demonstrated that SD impaired the learning and memory of rats and caused the decrease in endogenous H_2_S generation and the formation of excessive autophagy in the hippocampus of rats. These novel discoveries provide distinctive insights into understanding the mechanism underlying SD-induced cognitive impairment.

Exposure of SD has certain toxicity in nervous system of humans and animals (Novati et al., 2012; Basner et al., 2013). In agreement with this, sleep plays a key role in the removed of neurotoxic substances that produce in the waking state (Xie et al., 2013). Research has shown that SD impacts the expression and function of glutamate receptor (Ravassard et al., 2009) and neurogenesis (Meerlo et al., 2009) in the hippocampus. In addition, SD interferes with the hippocampal synaptic plasticity and hippocampal long-term potentiation (LTP), contributing to the deficit of cognition in animals and humans (Alkadhi et al., 2013). In our present study, SD-exposed rats showed cognitive impairment in Y maze test, OLT, NORT, and MWM test. Our results are consistent with the finding that exposure of rats with SD leads to the dysfunction of learning and memory (McDermott et al., 2006). Clinical studies show that SD leads to cognitive deficits (Olaithe et al., 2018). However, the molecular mechanisms underlying SD-induced cognitive impairment have not been fully clarified.

The biologic function of H_2_S mainly include antioxidation, anti-apoptosis and anti-inflammation for central nervous system (Wei et al., 2014), which imply the protective effect of H_2_S on neurodegenerative diseases. It has been reported that physiological concentration of H_2_S specifically enhances the activity of *N*-methyl-D-aspartate receptor and facilitates the hippocampal synaptic plasticity and LTP (Zhang and Bian, 2014; Kamat et al., 2015). Previous work has implicated that exogenous H_2_S attenuates diabetes-associated cognitive impairment (Tang et al., 2015; Ma et al., 2017) and has a protective effect from traumatic brain injury-induced cognitive impairment (Karimi et al., 2017; Ma et al., 2017). In addition, we have demonstrated that inhibition of H_2_S generation mediates homocysteine-induced cognitive impairment (Li M.H. et al., 2014), which is prevented by exogenous H_2_S (Li M. et al., 2017; Tang et al., 2018). Thus, we speculate that inhibited hippocampal H_2_S generation may be associated with the pathophysiology of SD-induced cognitive impairment in rats. In our present study, we demonstrated both the decreased expression of H_2_S-synthesizing enzyme (CBS and 3-MT) and the inhibition of H_2_S generation in the hippocampus of SD-exposed rats. Studies showed that the decreased activity of CBS mediates homocysteine- and formaldehyde-induced cognitive deficits (Tang X. Q. et al., 2013; Kumar et al., 2017). Likewise, the level of endogenous H_2_S is decrease in the animal model and the patients of Alzhermer’s disease (Liu et al., 2008; Liu H. et al., 2015; Karimi et al., 2017). Thus, it is reasonable to believe that inhibition of hippocampal H_2_S generation contributes to SD-induced cognitive impairment.

Autophagy is essential for maintaining metabolic balance by digestion of misfolded proteins and dysfunctional organelles (Mizushima and Komatsu, 2011; Shehata and Inokuchi, 2014). However, it has been reported that excessive activation of autophagy damages synaptic plasticity in hippocampus (Hao et al., 2018) and that excessive activation of autophagy in hippocampus is responsible for cognitive impairment induced by hypoxic-ischemic brain injury (Xu et al., 2016) and sevoflurane (Li X. et al., 2017). Interestingly, inhibition of autophagy has a protective effect on cognitive impairment (Wang et al., 2015, 2017; Kong et al., 2018). So we speculated that excessive autophagy in hippocampus is inseparable from cognitive impairment of SD-exposed rats. Our present study found that the protein expressions of Beclin-1 and LC3-II, which are important for regulation of autophagy, were remarkably upregulated in the hippocampus of SD-exposed rats, while the expression of P62 was significantly downregulated. Meanwhile, the number of autophagosome was also increased in the hippocampus of SD-exposed rats. These data demonstrated that SD induces excessive autophagy in the hippocampus of rats. Interestingly, previous studies showed that the expression of LC3-II was increased in sevoflurane induced cognitive impairment, while p62 was decreased (Li X. et al., 2017), which is consistent with our results. Based on the evidence that excessive autophagy in hippocampus is closely associated with cognitive impairment (Xu et al., 2016; Li X. et al., 2017; Hao et al., 2018), our present results suggested that excessive hippocampal autophagy is an important pathological mechanism involved in the SD-induced cognitive impairment.

## Conclusion

Taken together, the present study demonstrated that SD causes impairment in cognitive function, inhibition of hippocampal H_2_S generation, and excessiveness in hippocampal autophagy. We suggested that SD-caused cognitive impairment may be due to the decreased endogenous H_2_S generation and the excessive autophagy in hippocampus. Increasing studies have demonstrated that H_2_S inhibits excessive autophagy (Shui et al., 2016; Jiang et al., 2017). Therefore, we suggested that the inhibition of endogenous hippocampal H_2_S generation causes the formation of excessive autophagy in the hippocampus of SD-exposed rats. The limitation of this article is that we did not demonstrate whether cognitive impairment induced by SD is improved by the inhibitors of autophagy or exogenous H_2_S and that whether other brain regions have the same pathological changes. In the future, we will explore the changes of H_2_S generation and autophagy in other brain regions, investigate the protective actions of autophagy inhibitor and exogenous H_2_S in SD-induced cognitive impairment, and detect the levels of H_2_S in the clinical samples. Based on the neuroprotective role of H_2_S, our findings opened a novel avenue that H_2_S might be a potential agent for treatment of cognitive impairment induced by SD.

## Author Contributions

X-QT and WZ were responsible for the experimental design. X-QT supervised this study. S-QY, LJ, and FL analyzed the data. S-QY and LJ wrote the manuscript. S-QY, LJ, FL, H-jW, MX, PZ, C-YW, and Y-RX performed the experiments.

## Conflict of Interest Statement

The authors declare that the research was conducted in the absence of any commercial or financial relationships that could be construed as a potential conflict of interest.
